# Metabolomics Analysis of Phenolic Composition and Content in Five Pear Cultivars Leaves

**DOI:** 10.3390/plants13172513

**Published:** 2024-09-07

**Authors:** Huijun Jiao, Qiuzhu Guan, Ran Dong, Kun Ran, Hongwei Wang, Xiaochang Dong, Shuwei Wei

**Affiliations:** State Key Laboratory of Nutrient Use and Management, Shandong Institute of Pomology, Longtan Road No. 66, Taian 271000, China; jiaohj_njau@163.com (H.J.); guanqiuzhu@163.com (Q.G.); dryan2011@163.com (R.D.); rkrl001@126.com (K.R.); 15020812363@139.com (H.W.)

**Keywords:** pear, metabolomics, phenolic compound, antioxidant activity, DPPH

## Abstract

Phenolic compounds are the predominant chemical constituents in the secondary metabolites of plants and are commonly found in pears. In this study, we focused on the analysis of the phenolic composition and antioxidant activity of leaves from five pear cultivars (Cuiguan, Chaohong, Kuerle, Nanguoli, and Yali) and tea leaves (Fudingdabai as the control) using ultra-performance liquid chromatography coupled with electrospray ionization triple quadrupole mass spectrometry. The results indicated significant differences in the amount and composition of phenolic metabolites between tea and pear leaves, as well as among the five pear varieties. Only approximately one-third of the metabolites exhibited higher levels in pear leaves compared to that in tea leaves. The total phenol content in the Yali cultivar was higher than that in the other pear cultivars. Furthermore, specific phenolic metabolites with high expression were identified in the leaves of different groups. The levels of delphinidin 3-glucoside, aesculin, prunin, cosmosiin, quercetin 3-galactoside, isorhamnetin-3-O-glucoside, nicotiflorin, narcissin, chlorogenic acid, and cryptochlorogenic acid were relatively high among the five pear cultivars. (-)-Gallocatechin gallate, 6-methylcoumarin, aesculetin, hesperidin, kaempferol, and caftaric acid were identified as specific metabolic substances unique to each type of pear leaf. Most of the differential metabolites showed positive correlations and were primarily enriched in the flavonoid biosynthesis, flavone and flavonol biosynthesis, and phenylpropanoid biosynthesis pathways. DPPH (1,1-Diphenyl-2-picrylhydrazyl radical) analysis indicated that the Yali cultivar exhibited the highest antioxidant activity compared to other varieties. This systematic analysis of the differences in phenolic metabolite composition and antioxidant activity between pear and tea leaves provides a theoretical foundation for the development and utilization of pear leaf resources.

## 1. Introduction

Pear (*Pyrus* spp.) is one of the important economic fruits of *Rosaceae* and is widespread across the world. China is one of the origins of pears, with a long history of cultivation and a rich diversity of varieties. The main pear cultivars in China include *P. pyrifolia* (Burm. F.) Nakai, *P. ussuriensis* Maxim, *P. sinkiangensis* T. T. Yu, *P. communis* L., and *P. bretschneideri* Rehder [1]. Pears are rich in secondary metabolites, which play an important role in antioxidant activity and disease prevention. They have been used as a traditional remedy in China for more than 2000 years [2]. Pears are not only one of the most common edible fruits, but also serve as a herbal medicine for relieving cough, nourishing lung, alleviating constipation, and combating alcoholism [3]. There are three major groups of secondary metabolites based on their biosynthetic pathways: nitrogen-containing compounds (cyanogenic glycosides, alkaloids, and glucosinolates), phenolic compounds (flavonoids and phenylpropanoids), and terpenes (isoprenoids) [4]. Pears are abundant in various phenolic compounds, primarily quercetin, catechins, and chlorogenic acid, which exhibit strong antioxidant, anti-inflammatory, antiviral, and anticancer properties [5]. Studies have shown that every part of the pear tree, such as the root, bark, fruit, and leaf, contains phenolic compounds with notable antioxidant activities [6]. In pear fruit, the phenolic compounds mainly include hydroxycinnamic acid, hydroxyquinone, flavonol, and flavanol, among which chlorogenic acid, arbutin, catechin, epicatechin, and rutin are the main monomeric phenolic compounds [7,8]. Wang et al. [9] identified 73 types of phenolic compounds in different pear varieties, with significantly higher levels of chlorogenic acid. Cui et al. [10] and Lee et al. [11] reported that arbutin and chlorogenic acid are the predominant components of phenolic compounds in pear fruit. In addition, phenolic compounds also have different distribution patterns in pear fruit. Studies by Li et al. [12] revealed that the content of phenolic compounds is highest in the skin of pear fruits followed by the core part, while it is lowest in pulp. Andreotti et al. [13] found that among five European pear varieties, chlorogenic acid was mainly enriched in the core part of the fruit while being less abundant in both the skin and pulp. Furthermore, changes occur during the fruit growth development process as well. In Korean pear varieties, Park et al. [14] observed that total phenolic and flavonoid contents decreased as fruits matured, with the total amount in young fruits being 1.5 to 2.5 times higher than those in ripe fruits. While there has been a considerable focus on investigating the content and types of phenolic metabolites in pear fruits, research on pear leaves remains relatively limited. 

Phenolic metabolites play an important role in plant growth and development. The composition and variety of phenolic compounds are important evaluation indicators in fruit breeding. The concentrations of phenolic compounds are used to select breeding materials to improve apple scab resistance and enhance the nutritional value of fruits [15]. Red raspberries contain numerous polyphenolic compounds associated with reducing blood pressure, mitigating atherosclerotic development, and improving lipid profiles and vascular function [16]. In addition, research has demonstrated a correlation between antioxidant activity and phenolic compounds in pears by analyzing and comparing various pear varieties in the Netherlands. This correlation is primarily influenced by the presence of catechins and proanthocyanidin polymers [5]. A study conducted by Jiang et al. demonstrated that pear fruits at the young fruit stage (50 days after flowering) exhibit relatively elevated levels of phenolic compounds and antioxidant capacity [17]. Fruits from traditional Serbian pear varieties are rich in bioactive components and could be used as functional foods or for potential nutraceutical applications to prevent diseases induced by oxidative stress [18]. Therefore, it is necessary to conduct an investigation into the antioxidant properties of phenolic compounds in pear leaves and to further explore their potential applications.

At present, nearly 200,000 secondary metabolites have been identified in plants, and are categorized into various groups including terpenoids, alkaloids, phenolics, steroids, flavonoids, and tannins [19,20]. Secondary metabolites play an important role in plant responses to stress such as disease and insect resistance. Sulfur-containing metabolites (terpenes and flavonoids) and nitrogen-containing metabolites (alkaloids, cyanogenic glucosides, and non-protein amino acids) are involved in plant defense responses [21]. Furthermore, the bioactive compounds derived from secondary metabolism in plants may also confer various beneficial effects on humans and animals, so they have a great value in the diet [22]. Leaves, as crucial organs for plant photosynthesis, contain a diverse array of bioactive ingredients with potential implications for the food, health products, and pharmaceutical industries [23]. Leaves are also the source organs in the plant source–sink relationship, with many important intermediate products produced by primary metabolism serving as the basic substances for secondary metabolism in other organs. The leaves of tea tree [*Camellia sinensis* (L.) O.Kuntze] contain a high concentration of phenolic metabolites such as flavanols, anthocyanins, flavones, and flavonols, which possess potent antioxidant, anti-cancer, and blood-glucose-lowering effects [24]. Its new leaves can be processed into different types of tea with unique flavors after different types of processing [25]. The advancement in the exploration and application of phenolic compounds in tea leaves holds significant implications for the investigation of phenolic substances in pear leaves.

Metabolomics is an important technique for analyzing and studying the composition and content of secondary metabolites in plants. The changes in the concentrations of sugars, organic acids, and phenolic acids in Yali pears were tested using HPLC during storage [26]. In this study, to identify the content and types of phenolic substances in pear leaves, Cuiguan (CG), Chaohong (CH), Kuerle (KEL), Nanguo (NG), and Yali (YL) leaves were collected and analyzed for phenolic compounds using metabolomics, with the Fudingdabai (FDDB) tea leaves used as a control, as it is abundant in polyphenols, flavonoids, theanine, and other compounds. The aim of this study was to determine the composition and content of phenolic metabolites in pear leaves and to identify active substances or secondary metabolites with potential commercial value. This research provides a theoretical and scientific basis for the utilization of pear leaf resources.

## 2. Results

### 2.1. Qualitative Analysis of Metabolites in Leaves

**Figure 1:**
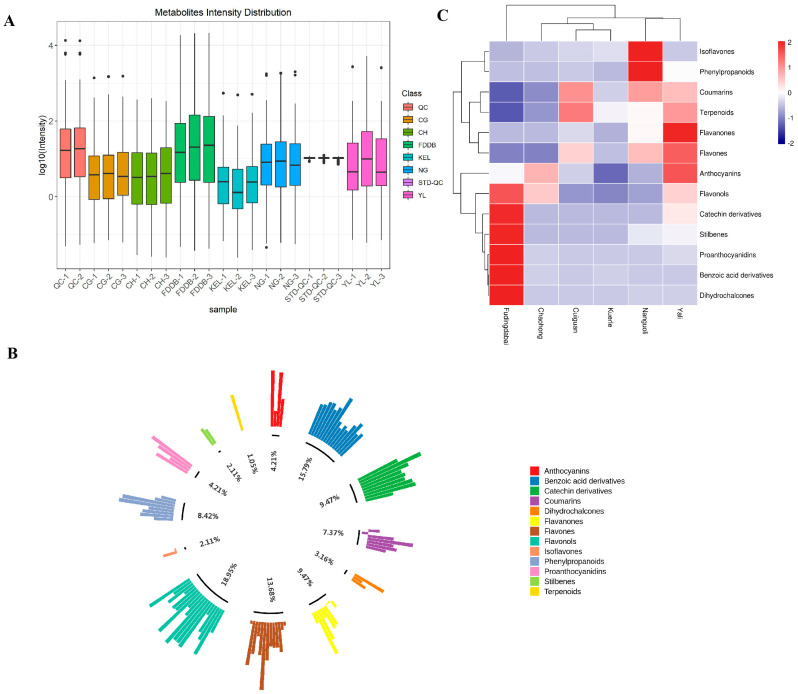
The density distribution of metabolites and analysis of metabolite cluster expression. (**A**): Sample metabolite strength box plot. (**B**): The expression classification of 13 phenolic substances detected. (**C**): HCA analysis of the expression level of 13 phenolic substances detected. Color scale represents log2 transformed expression content values. Blue indicates low expression and red indicates high expression.

In order to investigate the content and types of phenolic compounds in pear leaves, a metabolomics method was employed, with tea leaves used as the control. The results of the metabolite intensity distribution showed relatively concentrated distributions within each group (Figure 1A). A total of 130 phenolic substances was examined and categorized into 13 distinct classes, including anthocyanins, benzoic acid derivatives, catechin derivatives, coumarins, dihydrochalcones, flavanones, flavones, flavonols, isoflavones, phenylpropanoids, proanthocyanidins, stilbenes, and terpenoids. From the overall distribution trend of detected metabolites, we found that flavonols accounted for the highest proportion of metabolites (18.95%), followed by benzoic acid derivatives (15.79%) (Figure 1B). Additionally, we further analyzed the content levels of these categories, and the results revealed significant differences in the content of each category of phenolic compounds between pear and tea leaves. Specifically, pear leaves exhibited higher concentrations of anthocyanins, flavanones, flavones, terpenoids, coumarins, phenylpropanoids, and isoflavones. Conversely, tea leaves demonstrated elevated levels of benzoic acid derivatives, catechin derivatives, dihydrochalcones, flavonols, proanthocyanidins, and stilbenes (Figure 1C). Among the leaves of the six different pear cultivars, NG leaves were rich in isoflavones, phenylpropanoids, flavones, and coumarins; YL leaves were rich in coumarins, terpenoids, flavanones, flavones, anthocyanins, and flavonols; CG leaves showed enrichment in coumarins, terpenoids, and flavones; CH leaves mainly contained anthocyanins and flavonols; and KEL leaves did not show an obvious enrichment in secondary metabolite types (Figure 1C). The total content of phenolic compounds in YL was higher than in the other pear cultivars.

### 2.2. Quantitative Analysis and Trend Assessment of Metabolite Expression Levels

**Figure 2:**
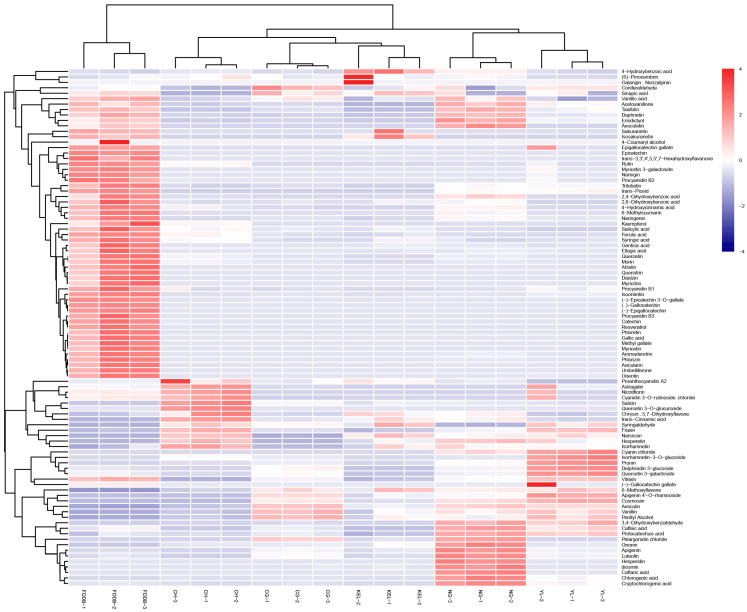
Hierarchical clustering analysis of metabolite expression level trend of six leaves. Color scale represents log2 transformed expression content values of each metabolite. Blue indicates low expression and red indicates high expression.

The quantitative analysis of the expression levels of all the metabolites selected from the six groups was conducted. A total of 130 phenolic substances were analyzed, with only 95 metabolites identified in our samples. The metabolites and their respective content information are shown in Table S1. A total of 66, 70, 70, 74, 70, and 84 metabolites were identified from CG, CH, KEL, NG, YL, and FDDB, respectively. We further analyzed the overall expression trends of the detected metabolites using a hierarchical clustering analysis (HCA). The results from the HCA revealed two distinct clusters, one comprising tea leaves and the other consisting of pear leaves, indicating that there are significant differences in the quantity and composition of phenolic metabolites between tea leaves and pear leaves (Figure 2). Our findings also demonstrated notable variations in the expression abundance of the metabolites across the six leaf samples, with each group exhibiting specific highly expressed metabolites. FDDB leaves showed a greater variety of highly expressed metabolites compared with the pear varieties. In more than half of the detected metabolites in FDDB leaves, the content was higher than in pear leaves. Most of these metabolites belonged to categories such as catechin derivatives, coumarins, flavanones, flavones, isoflavones, and phenylpropanoids. Only approximately one-third of the metabolites exhibited higher levels in pear leaves than in tea leaves, including ononin, 6-methoxyflavone, aesculin, isorhamnetin-3-*O*-glucoside, 4-hydroxybenzoic acid, syringaldehyde, vanillin, 3,4-dihydroxybenzaldehyde, trans-cinnamic acid, chlorogenic acid, prunin, fraxin, salicin, perillyl alcohol, and protocatechuic acid. In pear leaves, the top 10 metabolites with the highest average content included cosmosiin, epigallocatechin gallate, chlorogenic acid, delphinidin 3-glucoside, quercetin 3-galactoside, rutin, nicotiflorin, cyanidin 3-*O*-rutinoside chloride, cryptochlorogenic acid, and narcissin. There were 13 metabolites with higher levels in CH leaves, including proanthocyanidin A2, astragalin, nicotiflorin, and salicin. NG leaves had the highest number of highly expressed metabolites (approximately 35) among the six pear varieties, including unique highly expressed metabolites such as eriodictyol, hesperidin, acetovanillone, caftaric acid, pelargonidin chloride, and vanillic acid. Furthermore, CG, KEL, and YL leaves contained 13, 11, and 21 highly expressed metabolites, respectively (Figure 2).

Additionally, in comparison to FDDB, trans-cinnamic acid, 6-methoxyflavone, and ononin exhibited specific expression in pear leaves, with expression levels ranging from 83.43 to 521.71 ng/g, 1.45 to 3.98 ng/g, and 13.86 to 97.60 ng/g, respectively. Besides these, syringaldehyde, salicin, catechin derivatives, aesculin, prunin, isorhamnetin-3-*O*-glucoside, chlorogenic acid, and perillyl alcohol showed higher expression levels in pear leaves, with levels exceeding 457.07 ng/g, 2534.52 ng/g, 2743.69 ng/g, 17,548.35 ng/g, 7250.62 ng/g, 2402.63 ng/g, 50,306.17 ng/g, and 2280.85 ng/g, respectively. Additionally, only a few specific phenolic metabolites were detected in each variety of pear. Kaempferol was found to be uniquely expressed in CH, while sakuranetin and isosakuranetin were identified in KEL. Aesculetin, 6-methylcoumarin, hesperidin, and caftaric acid were the exclusive phenolic compounds observed in NG. (-)-Gallocatechin gallate, naringin, and myricetin 3-galactoside exhibited unique expressions in YL. These findings indirectly suggest the presence of diverse phenolic compounds in the leaves of various pear cultivars.

### 2.3. Screening and Analysis of Differential Metabolites

**Figure 3:**
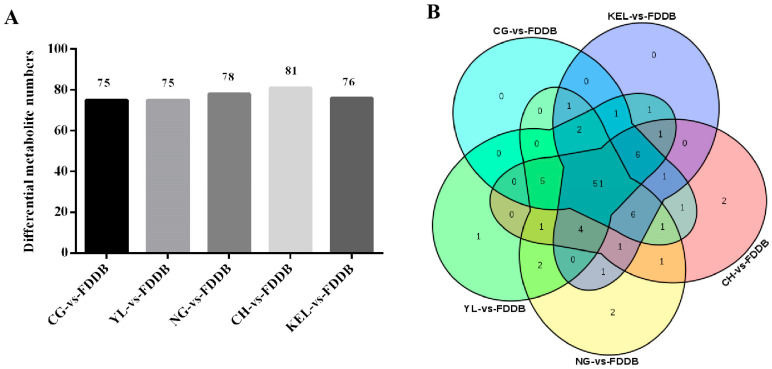
Comparative analysis of differential metabolites in five comparison groups. (**A**): Statistical analysis of the number of differential metabolites in five comparison groups. The different colors indicate different comparison groups. (**B**): Comparative analysis of differential metabolites by Venn diagram in five comparison groups. The different colors indicate different comparison groups.

Based on the results of the univariate analysis of the detected metabolites, differential metabolites were further selected using fold change (FC) and a *p*-value < 0.05 as criteria. Metabolites meeting both the criteria of FC > 2/FC < 0.5 and a *p*-value < 0.05 were considered differential metabolites. When comparing CG, YL, NG, CH, and KEL with FDDB, 75, 75, 78, 81, and 76 significantly different metabolites were detected in each group, respectively (Figure 3A). Additionally, in the CG-vs-FDDB, YL-vs-FDDB, NG-vs-FDDB, CH-vs-FDDB, and KEL-vs-FDDB comparison groups, 20, 24, 29, 21, and 18 metabolites showed upregulated expression levels, while 55, 51, 49, 60, and 58 metabolites showed downregulated expression levels, respectively. In comparison to FDDB leaves, pear leaves exhibited mainly upregulated metabolites including 3, 4-dihydroxybenzaldehyde, chlorogenic acid, ononin, perillyl alcohol, prunin, salicin, trans-cinnamic acid, and vanillin. The downregulated differential metabolites in pear leaves mainly included (-)-epigallocatechin, 4-hydroxycinnamic acid, afzelin, aromadendrin, daidzin, epicatechin, ferulic acid, gentisic acid, myricetin, naringenin, naringin, phloretin, phlorizin, quercetin, resveratrol, rutin, and salicylic acid. A Venn diagram was used to analyze the distribution of differential metabolites between the five comparison groups, and the results showed that there were 51 shared differential metabolites across all comparisons. Additionally, in the comparison groups of CH-vs-FDDB, NG-vs-FDDB, and YL-vs-FDDB, two, two, and one unique differential metabolites were identified, respectively.

### 2.4. K-Means Analysis of Differential Metabolites

**Figure 4:**
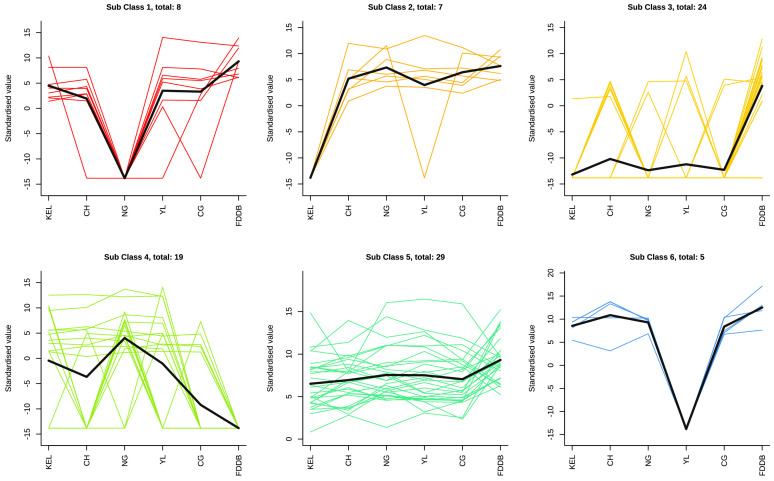
K-mean cluster analysis of differential metabolites. The black line shows the overall trend of metabolites in each sub class. The other different colored lines represent different sub classes.

To gain a more comprehensive understanding of the expression trends of the selected differential metabolites, we performed K-means clustering to analyze the overall expression patterns of the differential metabolites between KEL, CH, NG, YL, and CG leaves in comparison to FDDB leaves. All the differential metabolites were divided into eight subclusters, with clusters 4, 5, and 8 showing a higher number of differential metabolites. In subclusters 1 and 3, lower expression levels of metabolites were detected in NG leaves. Specifically, subcluster 1 contained syringaldehyde, gallocatechin, eriodictyol, delphinidin 3-glucoside, taxifolin, and daphnetin, which were classified as benzoic acid derivatives, catechin derivatives, flavanones, anthocyanins, flavonols, and coumarins. In subclusters 2 and 5, the metabolite expression levels presented an upregulated trend, with relatively lower expression levels in KEL leaves. These findings suggest that specific secondary metabolites with high expression are present in different types of leaves, which may contribute to variations in fruit quality. The expression levels of metabolites in YL leaves showed opposite trends between subclusters 6 and 7. Subcluster 6 was characterized by the presence of epigallocatechin gallate, nicotiflorin, astragalin, cyanidin 3-*O*-rutinoside-chloride, and vitexin as the predominant metabolites. In contrast, subcluster 7 was distinguished by the predominance of naringin, trans-piceid, myricetin 3-galactoside, cyanin chloride, and protocatechuic acid. In subclusters 3 and 6, most metabolite expression levels in CH leaves were relatively higher than those in the other leaves, and these metabolites included methyl gallate, afzelin, 2,4-dihydroxybenzoic acid, acetovanillone, coniferaldehyde, nicotiflorin, astragalin, and cyanidin 3-*O*-rutinoside-chloride (Figure 4). These results suggest that specific secondary metabolites with high expressions are present in different types of leaves.

### 2.5. KEGG Analysis of Differential Metabolites

**Figure 5:**
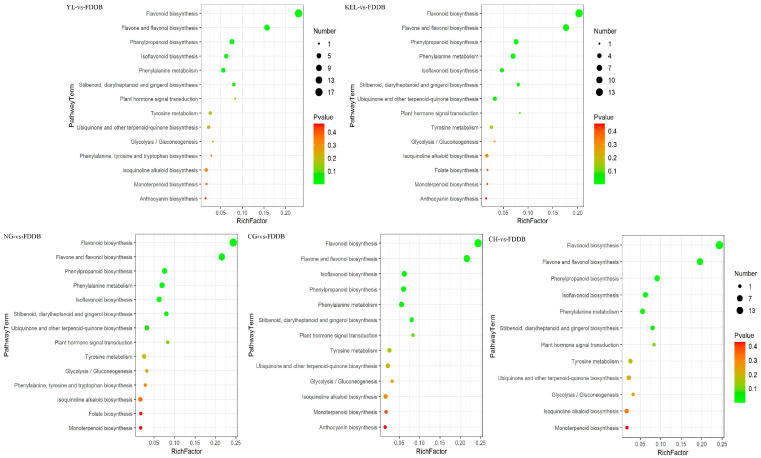
KEGG enrichment pathway analysis of differential metabolites in comparison groups. The greater the rich factor, the greater the degree of enrichment. The color from red to green indicates that the *p*-value decreases in turn. The larger the dot, the more metabolites are enriched in the pathway.

**Figure 6:**
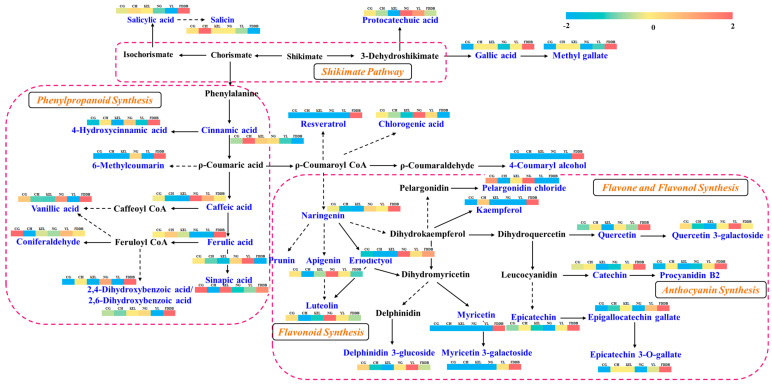
The metabolic pathway diagram for the main phenolic metabolites. The content of phenolic metabolites is shown in heat maps based on the abundance in the metabolite profile. Blue indicates low expression and red indicates high expression. The main phenolic metabolites detected in this study are colored in blue and their corresponding metabolic pathways are colored in yellow. The metabolites in black represent that they were not detected.

We further analyzed the KEGG pathways of the differential metabolites between CG, CH, KEL, NG, YL, and FDDB. Among the five comparison groups, a total of 15 significantly enriched KEGG pathways were identified. Specifically, 13, 12, 14, 14, and 14 enriched pathways were detected in the respective comparison groups (Figure 5). The differential metabolites were predominantly rich in flavonoid biosynthesis (pxb00941), flavone, and flavonol biosynthesis (pxb00944), isoflavonoid biosynthesis (pxb00943), phenylpropanoid biosynthesis (pxb00940), and phenylalanine metabolism (pxb00360) pathways. Notably, the KEGG enrichment pathways of differential metabolites exhibited similarities across all five comparison groups. There were 14~18 and 11~14 differential metabolites enriched in flavonoid biosynthesis and flavone and flavonol biosynthesis pathways, respectively. However, in the KEL-vs-FDDB and NG-vs-FDDB groups, the differential metabolites were also enriched in the folate biosynthesis (pxb00790) pathway. Only 4-hydroxybenzoic acid was enriched in this KEGG pathway, with an upregulated expression level. Subsequently, we constructed a metabolic pathway diagram for the main phenolic metabolites and labeled the changes for select relevant metabolites (Figure 6). It is evident that there are significant differences in the metabolic pathways of the six categories of leaves.

### 2.6. Differential Metabolite Correlation Analysis

**Figure 7:**
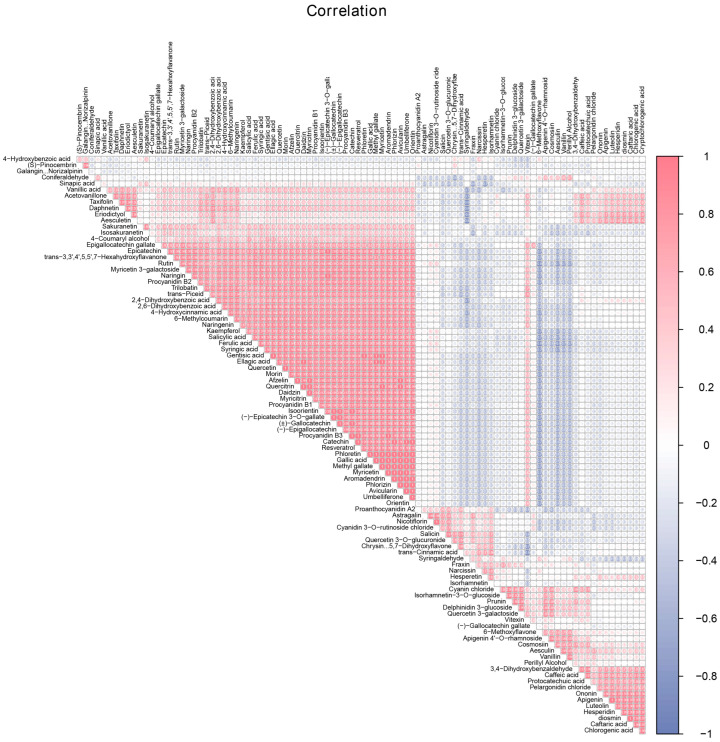
Correlation analysis of metabolites in the six groups of leaves. Blue indicates a negative correlation and red indicates a positive correlation.

Correlation analysis allows for the quantification of the degree of correlation between different metabolites, facilitating a deeper understanding of their interrelationship during changes in biological states. In this study, we used the Pearson correlation coefficient to assess the correlation between differential metabolites. First, we analyzed the overall correlation of metabolites and found that among the 95 metabolites detected, 2230 pairs of metabolites were positively correlated (r > 0), and 2235 pairs were negatively correlated (r < 0). Additionally, we focused on the correlation between different metabolites. The results showed that the majority of differential metabolites with high expression exhibited significant positive correlations. Vanillin had a positive correlation with aesculin, chlorogenic acid, caftaric acid, cryptochlorogenic acid, isorhamnetin-3-*O*-glucoside, and others. Conversely, vanillin showed a negative correlation with rutin, narcissin, cyanidin 3-*O*-rutinoside-chloride, and proanthocyanidin A2. Salicylic acid demonstrated a positive correlation with 6-methylcoumarin, aesculetin, aromadendrin, catechin, epicatechin, rutin, proanthocyanidin A2, procyanidin B1, procyanidin B2, and procyanidin B3. Additionally, salicylic acid exhibited a negative correlation with salicin, prunin, isorhamnetin-3-*O*-glucoside, narcissin, and others (Figure 7). These results suggest that the related differential metabolites may be involved in the same metabolic pathway and synergistically regulate plant growth and development.

### 2.7. Evaluation of Antioxidant Capacity of Leaves

**Figure 8:**
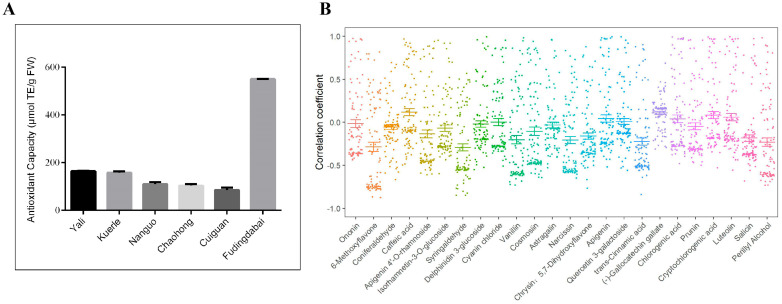
Evaluation and correlation analysis of the antioxidant capacity of leaves. (**A**): Detection of antioxidant capacity of six groups of leaves. The different colors indicate different sample groups. The values presented are the means of three independent replicates. (**B**): Correlation analysis of antioxidant capacity and metabolites in leaves. The different colors indicate different metabolites.

Antioxidant capacity is the most important property of phenolic compounds, arising from the synergistic action of these compounds. To explore the antioxidant activity of different leaves, the DPPH clearance rate was tested to reflect differences in antioxidant activity. The results showed that the antioxidant activity of tea leaves was stronger compared to those of the other five pear samples. Among the five pear varieties, YL leaves exhibited the highest antioxidant level, followed by KEL, NG, CH, and CG (Figure 8A). Furthermore, a correlation analysis between the phenolic metabolites present in the leaves and their antioxidant activity showed that caffeic acid, (-)-gallocatechin gallate, cryptochlorogenic acid, luteolin, apigenin, chlorogenic acid, quercetin 3-galactoside, and cyanin chloride were positively correlated with the antioxidant capacity of leaves, while ononin, delphinidin 3-glucoside, and other secondary metabolites were negatively correlated with antioxidant capacity (Figure 8B). The antioxidant activity of YL leaves was significantly higher than that of the other pear leaves. In YL leaves, the levels of (-)-gallocatechin gallate, cryptochlorogenic acid, and quercetin 3-galactoside were measured at 145,738.46 ng/g, 210,403.08 ng/g, and 1,334,555.38 ng/g, respectively. Furthermore, their contents were noticeably higher than those found in the other pear leaves, with the unique presence of (-)-gallocatechin gallate in YL leaves. These findings suggest that the aforementioned phenolic compounds are the primary contributors to the antioxidant activity of pear leaves.

## 3. Discussion

Metabolomics is widely employed for the isolation and identification of plant metabolites, effectively capturing information about plant metabolic pathways. Plant phenols, a heterogeneous group of secondary metabolites, are derived from the phenylpropanoid pathway in plants and contribute to the pigmentation of flowers, fruits, and vegetables [19]. FDDB tea is renowned for its rich phenolic content, including catechin, flavonoids, flavonol glycosides, anthocyanins, quercetin, kaempferol, and others. These compounds not only meet nutritional needs but also possess antioxidant, anti-inflammatory, antibacterial, anti-cancer, blood pressure-lowering, and blood-lipid-lowering effects. Therefore, FDDB tea was used as a reference to investigate the content and variations of phenolic metabolites in pear leaves. The results of this study indicated significant differences in the content and composition of phenolic metabolites between the leaves of five pear varieties and FDDB tea leaves, which may contribute to the distinct physiological functions of tea and pear leaves. Numerous metabolites synthesized in pear leaves not only fulfill the requirements for their growth but also provide essential nutrients for fruit maturation. In addition, many unique phenolic metabolites were identified in leaves of each pear variety, including kaempferol, sakuranetin, isosakuranetin, aesculetin, 6-methylcoumarin, hesperidin, caftaric acid, (-)-gallocatechin gallate, naringin, and myricetin 3-galactoside, many of which possess significant medicinal properties (Table S1). For instance, kaempferol exhibits anti-inflammatory, anti-diabetic, antioxidant, antibacterial, anti-tumor, neuroprotective, and cardioprotective activities, and is being applied in cancer chemotherapy. Specifically, food rich in kaempferol has been associated with a reduced risk of developing certain types of cancers, such as skin, liver, and colon cancer [27]. Aesculetin, a coumarin derivative, exhibits potent osteo-inductive properties by enhancing osteoblastogenesis and matrix-vesicle-mediated mineralization [28]. 6-Methylcoumarin displays potent antifungal activity against *Valsa mali* and plays a key role in preventing of apple Valsa canker through the inhibition of pathogenicity-related enzymes, including polygalacturonase, pectin lyase, and endo-1,4-β-D-glucanase [29]. Hesperidin, a natural flavonoid, has significant anticancer potential by modulating multiple pathways involving cell cycle arrest, apoptosis, anti-angiogenesis, anti-metastasis, and DNA repair in various cancer cells [30,31]. Caftaric acid, a hydroxycinnamic acid, plays a role not only in plant defense systems but also as signaling molecules for regulating plant development [32]. The anticarcinogenic activities of naringin and its aglycone naringenin have been shown to be exerted through several cell signal transduction pathways, which can also overcome multidrug resistance resulting from different defensive mechanisms in cancer [33]. Furthermore, leaf secondary metabolites exhibit anti-inflammatory, antioxidant, antitumor, and anticancer activities, making them potential sources for extracting related substances and promising candidates for developing anti-inflammatory drugs. Therefore, it is essential to develop specific extraction methods for phenolic metabolites and establish a processing flow for pear leaf tea to enhance the medicinal and tea value of pear leaves. Further studies are needed to investigate the medicinal application of phenolic metabolites in pear leaves, encompassing drug development stages, regulatory challenges, market influences, and comparisons with existing treatments.

The composition and content of phenolic compounds in pear fruits have been investigated to some extent. Escarpa et al. [34] and Salta et al. [35] analyzed the phenolic content in the fruit skin and pulp of different Western pear varieties and found significant differences in the phenolic content between the skin and pulp, with a richer variety of phenolic compounds present in the skin. Yuan et al. [36] and Zeng et al. [37] tested the mature fruits of different Oriental pear varieties, revealing discernible variations in the types and contents of phenolic compounds across different cultivars. In this study, the leaves of YL, CG, CH, KEL, and NG pear cultivars also exhibited significant variations in both the types and contents of phenolic metabolites. In pear fruit, chlorogenic acid, arbutin, (+/−) catechin, epicatechin, rutin, gallic acid, and quercetin 3-galactoside were found to be the main phenolic compounds [8,9,10,12]. Colaric et al. [38] conducted an analysis of the concentrations of eight phenolic metabolites in Williams pear leaves and reported chlorogenic acid was identified as a major phenolic metabolite, followed by rutin, epicatechin, catechin, vanillic acid, and syringic acid. A total of 95 phenolic metabolites were identified from leaves of five pear varieties in our study. In addition to the mentioned metabolites above, phenolic metabolites with elevated levels of expression were also identified, such as cosmosiin, epigallocatechin gallate, delphinidin 3-glucoside, nicotiflorin, cyanidin 3-*O*-rutinoside chloride, cryptochlorogenic acid, and narcissin. These results will provide the theoretical basis for the study of functional substances of pear leaves. It should be noted that arbutin has not been detected in pear leaves, possibly due to its difficulty in separation. In addition, each pear cultivar displayed a distinct profile of highly expressed phenolic metabolites, likely attributed to their diverse genetic backgrounds (Figure 2). These cultivars belong to the species *P. bretschnrideri*, *P. pyrifolia, P. communis, P. sinkiangensis, and P. ussuriensis,* respectively. Furthermore, the specific expression of some metabolites in leaves may be related to the suitable planting area for pear cultivation. For instance, NG is mainly distributed in the Liaoning province of China; therefore, its specific secondary metabolites may be linked to its cold resistance. Moreover, the contents of phenolic compounds also vary with the growth seasons, growth years, and environment due to modifications in the gene expression or their encoded protein activity involved in metabolic pathways. Therefore, in the future, it is necessary to combine metabolomics with genomics, transcriptomics, proteomics and other advanced technologies to reveal the molecular regulatory mechanisms of synthesis and the metabolism of active constituents from phenolic metabolites in pear leaves. These can provide important theoretical foundations and technical support for the improvement of pear varieties and the synthesis of secondary metabolites.

The antioxidant activity of plants is attributed to the synergistic action of phenolic substances, which is also the most important property of phenolic compounds. Currently, there is widespread attention on developing antioxidant foods using plant materials with high antioxidant activity, reflecting a novel trend in the exploitation of plant resources. Many studies have investigated the relationship between phenolic substances and antioxidant activity in pear fruit. Salta et al. [35] analyzed the phenolic extracts from five pear varieties using DPPH and found that Rocha exhibited a significantly higher antioxidant capacity than the other four varieties, recommending it as a valuable natural source of antioxidants. Zeng et al. [37] revealed a highly significant correlation between total flavonoid and total phenol content in pear fruits and antioxidant capacity, with variations observed among different varieties. Studies by Jiang et al. [17] and Wu et al. [39] demonstrated that the contents of arbutin, catechin, chlorogenic acid, quercetin rhamnoside, and kaempferol-3-*O*-rutinoside in Zaosu, Xinliqi, and Kuerle pear fruits were positively correlated with the antioxidant activity of the fruits. In this study, we also found that chlorogenic acid and quercetin 3-galactoside were positively correlated with the antioxidant capacity of pear leaves, and different pear varieties exhibited differences in leaf antioxidant activity as well. Notably, YL leaves exhibited significantly higher antioxidant activity than other pear leaves. YL is poised to serve as a promising germplasm resource for breeding high-antioxidant pear varieties by further investigating the genetic basis of leaf antioxidant capacity and identifying genotypes with high levels of antioxidant components.

## 4. Materials and Methods

### 4.1. Plant Material

The young leaves of five typical pear cultivars and tea were collected from pear and tea orchards at the Shandong Institute of Pomology. These included Yali (YL), Cuiguan (CG), Chaohong (CH), Kuerle (KEL), Nanguoli (NG), and Fudingdabai (FDDB). The leaves were immediately stored at low temperatures after being picked.

### 4.2. Metabolite Extraction

First, 50 mg of pear and tea leaf samples were weighed and placed into 2 mL EP tubes separately. An automated sample homogenizer (Model: JXF-STPRP-24/32, Shanghai Jingxin Industrial Development Co., LTD, Shanghai, China) with small stainless-steel beads was used to grind the samples at a frequency of 60 Hz for 2 min. Afterward, the samples were placed in an ice water bath and extracted using an ultrasonic cleaner (Model: SB-5200DT, Ningbo Xinzhi Biotechnology Co., LTD, Taizhou, China) for 20 min. The samples were centrifuged for 10 min (4 °C, 13,000× *g*), and 500 μL of the supernatant was transferred to a new EP tube. The above steps were then repeated once. A total of 800 μL of supernatant was collected. An amount of 100 μL of the supernatant was taken and evaporated, then reconstituted with 200 μL of water/methanol (*v*/*v* = 18:7) containing an internal standard (L-2-chlorophenylalanine) at a concentration of 12 ng/mL, and the sample was vortexed for 30 s and sonicated for 2 min. The sample was centrifuged for 5 min at 4 °C, 13,000× *g*, and 100 μL of the supernatant was collected. The supernatant was diluted 100-fold and both the diluted and original samples were transferred to LC amber vials and stored at −80 °C for further use. Additionally, quality control (QC) samples were prepared by mixing equal volumes of the extracted liquids from all samples. The volume of each QC sample was the same as that of the individual samples. The analytical instruments used in this study included a high-performance liquid chromatography system (Model: AB ExionLC, AB Sciex, Framingham, USA) and a triple quadrupole mass spectrometer (Model: Qtrap 6500+, AB Sciex, Framingham, MA, USA).

### 4.3. Chromatography Mass Spectrometry

In this study, an UPLC-ESI-MS/MS analysis was used to detect the target metabolites qualitatively and quantitatively. A metabolomics data analysis was conducted by Shanghai Luming Biotechnology Co., LTD. The chromatographic system used was a Waters ultra-high-performance liquid chromatograph. According to the properties of the phenolic substances, a Waters UPLC HSS T3 (100 × 2.1 mm, 1.7 μm) liquid chromatographic column was used. The sample size was 5 μL. The gradient elution system consisted of (A) water (containing 0.1% formic acid, *v*/*v*) and (B) acetonitrile (containing 0.1% formic acid, *v*/*v*) using the following gradients: 0.01 min, 5% B; 2 min, 5% B; 4 min, 30% B; 8 min, 50% B; 10 min, 80% B; 14 min, 100% B; 15 min, 100% B; 15.1 min, 5% B; and 16 min, 5% B. The flow rate was 0.35 mL/min, and the column temperature was 45 °C. All samples were kept at 4 °C during analysis. The mass ranged from 100 *m*/*z* to 1200 *m*/*z*. The scanning resolution of the primary mass spectrometry was 70,000, the scanning resolution of the secondary mass spectrometry was 17,500, and the collision energies were 10, 20, and 40 eV, respectively. The mass spectrometer operated as follows: spray voltage, 3800 V (+) and 3200 V (−); sheath gas flow, 35 arbitrary units; auxiliary gas flow, 8 arbitrary units; capillary temperature, 320 °C; aux gas heater temperature, 350 °C; and lens RF level, 50.

### 4.4. Qualitative and Quantitative Analysis of Metabolites

The default parameters of SCIEX OS-MQ 1.6.1 software (Sciex, Framingham, MA, USA) were used to automatically identify and integrate each MRM transition and assist in manual inspection. The stability of the instrument was evaluated by overlapping display and the analysis of the total ion flow diagram (TIC diagram) of the spectrum detection and the analysis of different quality control standards. The RSD value was calculated based on the data acquisition results (RSD ≤ 20). A mixed standard solution was prepared, and a gradient dilution of the mixed standard was carried out to obtain the corresponding quantitative mass spectrum data of the standard product at different concentrations. The standard curve for different metabolites was plotted with the concentration of the standard product (ng/mL) on the horizontal axis and the peak area of the mass spectrum peak on the vertical axis. The mass spectrum peak of each metabolite detected in different samples was manually corrected to ensure qualitative and quantitative accuracy. The peak area of each chromatographic peak represented the relative content of the corresponding metabolite. The integrated peak area of the metabolite was entered into the linear equation of the standard curve for calculation. Finally, the absolute content data of each metabolite in the actual sample were obtained.

### 4.5. Differential Metabolite Screening

Student’s t-test and a fold change analysis were frequently used to compare the differential metabolites between the two groups. A combination of the *p*-value and log2 (FC) was used to screen differential metabolites between groups. Generally, metabolites with *p* < 0.05 and an absolute log2 (FC) > 1 were considered differential metabolites.

### 4.6. Heatmap and Venn Analysis

According to the quantitative expression results of the metabolites, the data were standardized (log2), and the information on differential metabolites was screened by differential comparison groups. Then, heatmap and Venn analyses were performed using Metware Cloud, a free online platform for data analysis (https://cloud.metware.cn, accessed on 12 September 2023).

### 4.7. K-Means Analysis

The expression trends of different metabolites in six kinds of leaves were analyzed by a clustering analysis. The content value of each metabolite was selected, and Metware Cloud platform 2.0 (https://cloud.metware.cn/#/home, accessed on 15 September 2023) online software was used to filter out differential metabolites in the K-means cluster analysis. Due to the wide range of differences in metabolite content, the values of metabolite content were numerically converted, logarithms (log2) were taken, a manually specified clustering method was selected, and the classification number was set to 8.

### 4.8. KEGG Analysis

According to the KEGG (https://www.kegg.jp/, accessed on 20 October 2023) platform, an enrichment analysis of the differential metabolites was carried out. A pathway enrichment analysis was performed using the KEGG ID of differential metabolites, and the enrichment results of metabolic pathways were obtained. A hypergeometric test was applied to identify the pathway entries that were significantly enriched in differentially expressed metabolites compared with the whole background.

### 4.9. DPPH (1,1-Diphenyl-2-picrylhydrazyl Radical) Analysis

The determination of the DPPH clearance rate was based on Azevedo et al.’s method [40] with slight adjustments. An amount of 0.1 g of leaves was collected and ground into a homogenate at low temperature. A total of 1 mL of 80% methanol extract was added to the ground material and transferred to an EP tube. The samples were centrifuged at 12,000× *g* at 4 °C for 10 min, and the supernatant was retained and kept on ice for measurement. Then, 150 μL of the above extraction solution and 150 μL of the working solution were added to a new EP tube, mixed well, and left to stand at room temperature for 30 min in the dark. The mixed solution was centrifuged at 12,000× *g* for 5 min. Subsequently, 200 μL of the supernatant was taken into a 96-well plate, and the absorption value A was measured at 517 nm. Control and blank experimental groups were set up. The specific calculation formula was as follows: DPPH free radical clearance (%) = [(1 − (A _Sample_ − A _CK_)/A _blank_) ×100]%. The DPPH clearance rate was expressed as Trolox equivalent per gram of fresh sample, denoted as μmol TE/g. Each sample was measured three times for repeatability.

## 5. Conclusions

In this study, the phenolic content and composition of leaves from six groups were identified through a metabolomics analysis. The results confirmed significant differences between tea and pear leaves, as well as among the five pear varieties. Chlorogenic acid, cosmosiin, and epigallocatechin gallate were identified as major phenolic metabolites in pear leaves with the highest average content, followed by delphinidin 3-glucoside, quercetin 3-galactoside, rutin, nicotiflorin, cyanidin 3-*O*-rutinoside chloride, cryptochlorogenic acid, and narcissin. Furthermore, each pear cultivar displayed significantly high or low expression levels of specific metabolites with certain unique phenolic metabolites being identified, including kaempferol, sakuranetin, isosakuranetin, aesculetin, 6-methylcoumarin, hesperidin, caftaric acid, (-)-gallocatechin gallate, naringin, and myricetin 3-galactoside. These metabolites possess the potential to serve as valuable active substances or secondary metabolites with commercial applications. Notably, YL leaves exhibited significantly higher antioxidant activity compared to other pear leaves, suggesting the potential of YL pears as valuable parental material for breeding high-antioxidant varieties. Therefore, in order to fully explore the medical and commercial potentials of pears, we should enhance research on phenolic substances in pear leaves for medicinal and healthcare applications in the future, as well as for the development of novel sustainable food products. The systematic analysis presented here provides a theoretical foundation for the development and utilization of pear leaf resources.

## Data Availability

Data are contained within the article. The raw data supporting the conclusions of this article will be made available upon reasonable request.

## Supplementary Materials

The following supporting information can be downloaded at: https://www.mdpi.com/article/10.3390/plants13172513/s1. Table S1. The content of phenolic substances detected in pears and tea leaves (ng/g).
